# Preliminary Study of Right Ventricular Dyssynchrony Under High-Altitude Exposure: Determinants and Impacts

**DOI:** 10.3389/fphys.2020.00703

**Published:** 2020-07-02

**Authors:** Yuanqi Yang, Chuan Liu, Jingdu Tian, Xiaohan Ding, Shiyong Yu, Shizhu Bian, Jie Yang, Zhexue Qin, Jihang Zhang, Jingbin Ke, Fangzhengyuan Yuan, Chen Zhang, Rongsheng Rao, Lan Huang

**Affiliations:** ^1^Institute of Cardiovascular Diseases of PLA, The Second Affiliated Hospital, Third Military Medical University (Army Medical University), Chongqing, China; ^2^Department of Cardiology, The Second Affiliated Hospital, Third Military Medical University (Army Medical University), Chongqing, China; ^3^Department of Geriatric Cardiology, Chinese PLA General Hospital, Beijing, China; ^4^Department of Medical Ultrasonics, The Second Affiliated Hospital, Third Military Medical University (Army Medical University), Chongqing, China

**Keywords:** high altitude, right ventricular dyssynchrony, hypoxemia, pulmonary artery pressure, speckle-tracking echocardiography

## Abstract

The aims of this study were to explore the effect of high-altitude (HA) exposure on the incidence, determinants, and impacts of right ventricular dyssynchrony (RVD). In our study, 108 healthy young men were enrolled, and physiological and echocardiographic variables were recorded at both sea level and 4,100 m. By using two-dimensional speckle-tracking echocardiography, RVD was evaluated by calculating the R–R interval-corrected standard deviation of the time-to-peak systolic strain for the four mid-basal RV segments (RVSD4) and defined by RVSD4 > 18.7 ms. After HA exposure, RVSD4 was significantly increased, and the incidence of RVD was approximately 32.4%. Subjects with RVD showed lower oxygen saturation (SaO_2_) and RV global longitudinal strain and higher systolic pulmonary artery pressure than those without RVD. Moreover, myocardial acceleration during isovolumic contraction was increased in all subjects and those without RVD, but not in those with RVD. Multivariate logistic regression revealed that SaO_2_ is an independent determinant of RVD at HA (odds ratio: 0.72, 95% CI: 0.56–0.92; *P* = 0.009). However, the mean pulmonary artery pressure was linearly correlated with the magnitude of RVD in the presence of Notch. No changes were found in RV fractional area change, tricuspid annular motion, or tricuspid *s*’ velocity between subjects with and without RVD. Collectively, we demonstrated for the first time that HA exposure could induce RVD in healthy subjects, which may be mainly attributed to the decline in SaO_2_ as well as RV overload; the incidence of RVD was associated with reduced RV regional function and blunted myocardial acceleration.

## Introduction

Traveling to a high altitude (HA) for sports, work, or recreational purposes is becoming popular but poses some physiological challenges to the cardiovascular system, leading to hypoxic pulmonary vasoconstriction, pulmonary vascular remodeling, and increases in pulmonary artery pressure (PAP) (Xu and Jing, 2009). In turn, elevated right ventricular (RV) afterload may predispose the RV to remodeling, hypertrophy, or even failure if HA hypoxia is sustained (Penaloza and Arias-Stella, 2007). Although it has been proposed that RV systolic function is well preserved and that its diastolic function is sometimes altered under acute HA hypoxia (Naeije, 2010), exposure to HA at 8,000 m or acute hypoxia results in RV dilatation, an increased RV Tei index, and a decreased RV free wall longitudinal systolic strain (Kurdziel et al., 2017; Netzer et al., 2017). Moreover, significant decreases in tricuspid annular plane systolic excursion (TAPSE) and RV global longitudinal strain (RVGLS) have been found in healthy lowlanders after ascending to an HA of 5,050 m, suggesting that RV longitudinal systolic function is impaired (Stembridge et al., 2014). Therefore, although elevated PAP and HA hypoxia may potentially threaten RV performance, the effects of acute HA exposure on RV global or regional functions are controversial and inconclusive.

Evaluation of RV global or regional function at HA is of considerable importance because RV function (such as the Tei index) and elevated PAP are associated with impaired exercise capacity (Naeije et al., 2010; Yang et al., 2015). Moreover, a previous report showed that subjects with previous HA pulmonary edema developed HA right-heart failure with severe tricuspid regurgitation (TR), dilatation of the RV chamber, and RV postsystolic shortening (Huez et al., 2007). The latter reflects RV dyssynchrony (RVD), which was calculated by the R–R interval-corrected standard deviation (SD) of the time-to-peak systolic strain for the four mid-basal RV segments (RVSD4) as detected by speckle-tracking echocardiography (STE). The presence of RVD usually suggests the variability of time differences in regional contraction through RV strain curves and is a more accurate estimate of RV function than the tissue Doppler method (Murata et al., 2017). In patients with pulmonary hypertension (PH), the presence of RVD is always associated with the symptomatology, functional state, exercise performance, and clinical outcomes (Murata et al., 2017; Rehman et al., 2018). Recent evidence also showed that RVD occurred during hypoxia but not during exercise, suggesting the combined contributions of mechanical (RV afterload) and systemic (hypoxia) factors (Pezzuto et al., 2018). Based on these reasons, we hypothesize that RVD is present under acute HA exposure and can be explained by HA hypoxia or increased RV afterload.

We therefore conducted the present study to determine whether acute HA exposure can induce regional heterogeneity of RV contraction or RVD and to explore the determinants and impacts.

## Materials and Methods

### Participants and Study Procedures

Our research was approved by the Clinical Research Ethics Board of the Third Military Medical University (Army Medical University) (No. 2012015), was registered with the Center of Chinese Clinical Trial Registration (No. ChiCTR-RCS-12002232)^1^, and complied with the Declaration of Helsinki. Informed consent was obtained from each participant in our study. Young Chinese male soldiers who permanently lived in lowland areas were enrolled in 2013 at sea level (SL) (<500 m) in Chongqing and experienced a stair-type ascension to 4,100 m (Litang, Sichuan, China) from 400 m (Yanggongqiao, Chongqing, China) by bus within 7 days. Data collection was performed at SL and 5 ± 2 h after arrival at 4,100 m. Exclusion criteria were known cardiovascular and pulmonary diseases, any cardiovascular and pulmonary therapy, hematologic diseases, malignant tumors, liver or kidney dysfunction, history of HA-related diseases, or exposure to 2,500 m above SL in the past 6 months.

### Echocardiographic Assessments

An echocardiographic study was performed using a CX50 ultrasound system (Philips Ultrasound System, Andover, MA, United States) equipped with a 2.5-MHz adult transducer by registered sonographers according to a standardized protocol (Lang et al., 2015). Standard two-dimensional (2D) and Doppler images were obtained in the left lateral decubitus position after 10 min of rest, stored for three consecutive beats, and then analyzed off-line on a digital image analysis system (QLAB 10.5, Philips Healthcare, Andover, MA, United States) by two independent investigators in a blind fashion.

The following parameters were analyzed or calculated: right atrial (RA) area, RV end-diastolic area (RVEDA), RV end-systolic area (RVESA), and RV fractional area change [RV FAC, (RVEDA − RVESA)/RVEDA × 100]. Pulsed Doppler RV inflow recordings were performed in the apical four-chamber view. The peak early (*E*) and late (*A*) diastolic flow velocities were measured, and the *E*/*A* ratio was calculated from the transtricuspid inflow. From a standard apical four-chamber window, tricuspid annular motion (TAM) was measured to evaluate RV longitudinal function according to guidelines from the American Society of Echocardiography (Rudski et al., 2010).

Pulsed-wave Doppler recordings of pulmonary flow were performed from a modified basal short-axis view. Peak pulmonary artery velocity (PAV), the time of the pre-ejection period (PEP), pulmonary artery acceleration time (PAAT), pulmonary artery ejection time (PAET), and the presence of Notch were measured accordingly (Vonk Noordegraaf et al., 2015). The mean PAP (mPAP) was calculated from PAAT because it was present in all subjects; moreover, it is closely correlated with and substantially more feasible to measure than the maximal velocity of the TR jet (TRV) for the estimation of PAP: when PAAT was more than 120 ms, mPAP = 79 − (0.45 × AT), and when PAAT was less than 120 ms, mPAP = 90 − (0.62 × AT) (Wierzbowska-Drabik et al., 2019). Systolic PAP (SPAP) was calculated by the modified Bernoulli equation: 4 × TRV^2^ + 5 mmHg (an estimated central venous pressure) (Rudski et al., 2010).

With the use of color tissue Doppler imaging (TDI), the peak systolic velocity (tricuspid *s*’), early diastolic velocity (tricuspid *e*’), isovolumetric velocity (tricuspid IVV), and isovolumetric acceleration time (tricuspid IVAT) were measured at the lateral and septal tricuspid annular, and isovolumetric accelerated velocity (tricuspid IVA = IVV/IVAT) was calculated (Rudski et al., 2010).

### 2D STE

**FIGURE 1:**
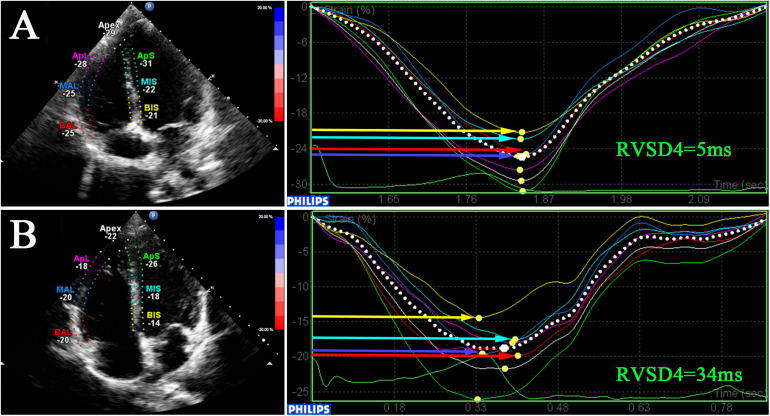
Dyssynchrony evaluation by speckle-tracking analysis. 2D speckle-tracking imaging in the RV-focused view in a participant at SL **(A)** and a participant at HA **(B)**. Curves represent longitudinal strain curves, which were used to measure RVD and contractile function. RV, right ventricular; RVSD4, SD of the time-to-peak systolic strain for the four mid-basal RV segments.

For STE analysis (QLAB 10.5), standard grayscale 2D images in the RV-focused apical four-chamber view were obtained (frame rate >60 fps) and digitally stored for three consecutive cardiac cycles. Speckles were tracked frame by frame throughout the RV wall automatically, and segments that failed to track were manually adjusted by the researchers. Any segment with persistently inadequate tracking was excluded. The peak systolic myocardial strain measured by 2D STE was assessed in six RV segments including 2D strain (2DS) basal, middle, apical (the average of septum and free wall), and RVGLS. The time-to-peak systolic strain was measured as the time from onset of QRS complexes on ECG to maximum RV shortening by strain. The SD of the time-to-peak systolic strain was calculated as a parameter of mechanical dyssynchrony in a model with four mid-basal RV segments according to Bazett’s formula; this measurement was called RVSD4 (Figure 1A). Using the upper 95% limit of young healthy subjects at SL in our study, a cutoff value of 18.7 ms was defined as the criterion for RVD (Badagliacca et al., 2015).

### Reproducibility

**FIGURE 2:**
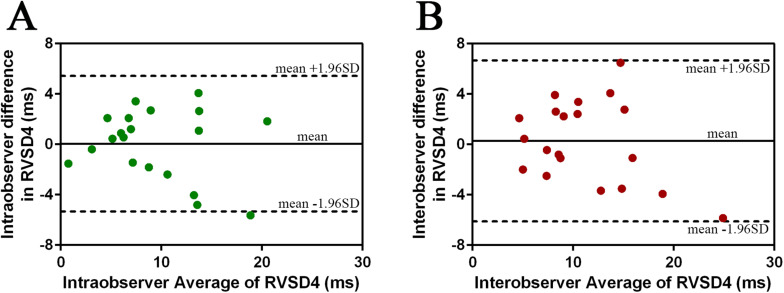
Intraobserver and interobserver variability in RVSD4 measurements assessed by the Bland–Altman method. **(A)** Intraobserver variability. **(B)** Interobserver variability. Abbreviations as in Figure 1.

Observer reliability of RVSD4 was assessed in 20 randomly selected subjects: 10 from SL and 10 from HA. Interobserver variability was analyzed by two separate observers. Assessments of intraobserver variability were conducted in a random order at least 1 week apart. The Bland–Altman method was used to evaluate the intraobserver variability [0.05 ± 2.74 (95% CI: −5.33 to + 5.43)] and interobserver variability [0.28 ± 3.26 (95% CI: −6.12 to + 6.68)], which is considered acceptable for our study (Figures 2A,B). The observer variability for the other echocardiographic parameters is presented in Supplementary Table 1.

### Covariates

Age, height, weight, Han ethnic status, and smoking status were obtained by using interviews or physical examinations. Body mass index (BMI) (kg/m^2^) was calculated by weight (kg)/height^2^ (m^2^). Heart rate (HR) was recorded from synchronous ECG when echocardiography was performed. Oxygen saturation (SaO_2_) was measured with warmed hands after signal stabilization by finger-pulse oximetry (Nonin ONYX OR9500, United States). Systolic and diastolic blood pressure (SBP and DBP) levels were taken in the supine position after 10 min of rest by an Omron HEM-6200 (Japan). Venous blood samples were collected after a 5-min rest, and routine blood tests were performed at SL and HA for the assessment of hemoglobin (Hb) concentration and hematocrit by a BC-3000 plus automated hematology corpuscle analyzer (Shenzhen, China).

### Statistical Analysis

SPSS 22.0 software (IBM Corp., Armonk, NY, United States) was used to perform all statistical analyses. Continuous data were represented as the mean ± SD or medians (25th, 75th percentiles) based on their normality distribution according to the Kolmogorov–Smirnov test, and categorical data were represented as count and percentage. Differences between groups were tested by using an independent-sample *t*-test or the Mann–Whitney *U* test for unpaired variables. The chi-square test was used for categorical variables. A paired *t*-test or the Wilcoxon test was used for comparisons of continuous variables between SL and HA. The risk factors for the incidence of RVD at HA were analyzed by univariate logistic regression analysis followed by a stepwise algorithm (*P* < 0.20 for entry and *P* < 0.05 for stay). Relationships between continuous variables were measured by linear regression analysis. The figures in this study were made by using GraphPad Prism 6 software (GraphPad Prism Software Inc., La Jolla, CA, United States). All tests were two-sided, and *P*-values <0.05 were considered statistically significant.

## Results

### Basic Characteristics and Physiological Parameters

**FIGURE 3:**
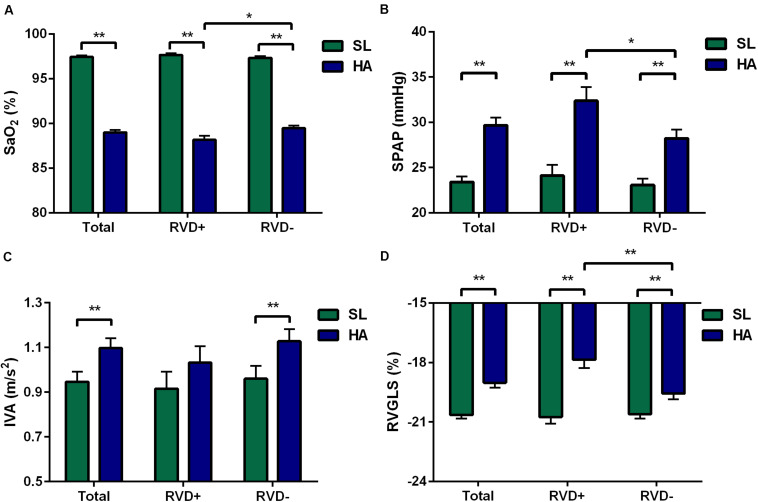
Differences in the total, RVD+, and RVD− subgroups. Different levels of SaO_2_ **(A)**, SPAP **(B)**, IVA **(C)**, and RVGLS **(D)** in the total, RVD+, and RVD− subgroups. Values represent the mean ± standard error of the mean. *P < 0.05, **P < 0.01. Abbreviations are as in Tables 1–3.

In this preliminary study, a total of 108 subjects aged 20 (19, 22) years with sufficient quality echocardiographic images were included. Representative pictures showed that RVSD4 was increased after acute HA exposure (Figures 1A,B). Using the cutoff value of 18.7 ms as the criterion for RVD, the incidence of RVD at HA was 32.4%. There were no significant differences between the RVD− and RVD + groups in age, BMI, Han ethnicity, or smoking status. After acute HA exposure, HR, SBP, DBP, Hb concentration, and hematocrit were increased, but the values of SaO_2_ decreased in all RVD− and RVD + groups. However, subjects in the RVD + group had a lower SaO_2_ [88.2 ± 2.7 vs. 89.0 ± 2.7%, *P* < 0.05] than those in the RVD- group after acute HA exposure (Table 1 and Figure 3A).

**TABLE 1:** Physiologic parameters of the participants at sea level and at high altitude.

**Variables**	**Total (*n* = 108)**			**RVD- (*n* = 73)**			**RVD + (*n* = 35)**		
	**SL**	**HA**	***P* value**	**SL**	**HA**	***P* value**	**SL**	**HA**	***P* value**
Age, years	20 (19, 22)		–	21 (19, 23)		–	20 (19, 21)		–
BMI, kg/m^2^	21.1 ± 1.8		–	21.0 ± 1.7		–	21.2 ± 2.0		–
Han ethnicity, *n* (%)	94 (87.0)		–	63 (86.3)		–	31 (88.6)		–
Smoking status, *n* (%)	69 (63.9)		–	47 (64.4)		–	22 (62.9)		–
HR, beats/min	66 (59, 74)	73 (64, 80)	<0.001	64 (58, 72)	71 (64, 78)	<0.001	70 (62, 77)	77 (64, 83)	0.003
SaO_2_,%	98 (97, 98)	89 (88, 91)	<0.001	98 (97, 98)	90 (88, 91)	<0.001	98 (97, 98)	88 (86, 91)*	0.009
SBP, mmHg	112 (106, 118)	120 (112, 127)	<0.001	111 (106, 118)	120 (113, 126)	0.035	116 (105, 124)	118 (112, 128)	0.006
DBP, mmHg	66 (62, 73)	78 (73, 84)	<0.001	66 (63, 73)	78 (73, 84)	<0.001	65 (60, 73)	78 (74, 85)	<0.001
Hb concentration, g/L	151.0 (142.0, 160.0)	167.0 (163.0, 177.5)	<0.001	149.0 (141.5, 159.5)	169.0 (162.5, 178.5)	<0.001	155.0 (144.0, 163.0)	167.0 (163.0, 173.0)	<0.001
Hematocrit (%)	42.5 (40.8, 45.3)	47.4 (44.7, 51.7)	<0.001	42.1 (40.7, 45.0)	47.9 (44.8, 52.4)	<0.001	43.6 (41.2, 46.1)	47.7 (44.7, 50.5)	<0.001

Values are the median (25th to 75th quartiles) or the mean ± SD.*P < 0.05 compared with RVD-. RVD, right ventricular dyssynchrony; SL, sea level; HA, high altitude; BMI, body mass index; HR, heart rate; SaO_2_, oxygen saturation; SBP, systolic blood pressure; DBP, diastolic blood pressure; Hb, hemoglobin.

### RV Function

After acute HA exposure, RVSD4 was significantly increased in all subjects [7.8 (2.9, 11.0) vs. 13.9 (6.7, 20.7) ms, *P* < 0.001] and in subjects with RVD [7.2 (3.3, 9.8) vs. 22.7 (20.9, 30.9) ms, *P* < 0.001]. In all subjects, RA area, RV EDA, RV FAC, TAM, tricuspid *E*/*A* ratio, RVGLS, and 2DS (basal, middle, and apical) were decreased, whereas tricuspid IVV and IVA were increased following acute HA exposure. However, RVGLS [−18.6 (−19.5, −16.6) vs. −19.6 (−21.3, −18.5)%, *P* < 0.01] and 2DS basal [−18.6 (−19.8, −15.9) vs. −19.0 (−20.4, −17.9)%, *P* < 0.05] were even lower in subjects with RVD than in those without RVD. Moreover, increases in tricuspid IVV and IVA were found in subjects without RVD but not in those with RVD (Table 2 and Figures 3C,D).

**TABLE 2:** Right ventricular parameters of the participants at sea level and at high altitude.

**Variables**	**Total (*n* = 108)**			**RVD- (*n* = 73)**			**RVD + (*n* = 35)**		
	
	**SL**	**HA**	***P* value**	**SL**	**HA**	***P* value**	**SL**	**HA**	***P* value**
RA EDA, cm^2^	11.7 ± 2.1	11.2 ± 2.4	**0.008**	11.7 ± 2.3	11.0 ± 2.4	**0.023**	11.8 ± 1.8	11.5 ± 2.4	0.457
RV EDA, cm^2^	22.4 ± 3.9	21.1 ± 3.6	**0.001**	22.3 ± 3.7	20.8 ± 3.4	**0.001**	22.6 ± 4.3	21.7 ± 4.0	0.218
RV ESA, cm^2^	12.4 ± 2.2	12.4 ± 2.4	0.921	12.4 ± 2.1	12.3 ± 2.3	0.665	12.4 ± 2.4	12.7 ± 2.7	0.602
RV FAC,%	44.3 (42.0, 47.7)	41.8 (37.5, 44.2)	**<0.001**	44.1 (42.2, 47.5)	41.6 (37.4, 44.3)	**<0.001**	45.6 (41.8, 48.2)	41.8 (38.6, 44.2)	**<0.001**
TAM, mm	20.6 (19.3, 23.0)	19.0 (17.3, 21.0)	**<0.001**	20.4 (19.1, 23.0)	18.9 (17.4, 21.0)	**<0.001**	21.0 (20.1, 23.2)	19.5 (16.8, 21.0)	**<0.001**
Tricuspid *E*/*A* ratio	1.99 (1.56, 2.39)	1.75 (1.41, 2.26)	**0.023**	2.02 (1.56, 2.48)	1.85 (1.47, 2.33)	0.068	1.98 (1.54, 2.17)	1.65 (1.36, 1.96)	0.238
Tricuspid *s*’, cm/s	7.9 ± 1.4	7.7 ± 1.3	0.279	7.9±1.4	7.6±1.3	0.160	7.7±1.4	7.8±1.4	0.813
Tricuspid *e*’, cm/s	7.3 ± 1.9	7.5 ± 2.2	0.233	7.5±1.8	7.6±2.2	0.850	6.8±2.2	7.4±2.1	0.247
Tricuspid IVV, cm/s	3.4 (2.0, 4.6)	4.1 (2.9, 5.0)	**<0.001**	3.7 (2.0, 4.5)	4.2 (3.0, 5.2)	**0.002**	2.8 (2.1, 4.7)	3.5 (2.5, 4.7)	0.133
Tricuspid IVAT, ms	37 (33, 42)	37 (33, 43)	0.970	38 (33, 43)	38 (33, 44)	0.725	36 (32, 40)	35 (30, 42)	0.632
Tricuspid IVA, cm/s^2^	0.90 (0.58, 1.25)	1.05 (0.76, 1.35)	**0.001**	0.91 (0.56, 1.25)	1.08 (0.77, 1.43)	**0.004**	0.86 (0.60, 1.34)	0.95 (0.74, 1.28)	0.114
RVGLS,%	−20.5 (−22.0, −19.3)	−19.2 (−20.3, −17.7)	**<0.001**	−20.5 (−21.9, −19.3)	−19.6 (−21.3, −18.5)	**0.008**	−20.1 (−22.1, −19.6)	−18.6 (−19.5, −16.6)**	**<0.001**
2DS basal,%	−20.0 (−21.3, −18.6)	−18.2 (−20, −15.4)	**<0.001**	−19.8 (−21.4, −18.6)	−19.0 (−20.4, −17.9)	**0.038**	−20.1 (−21.3, −18.6)	−18.6 (−19.8, −15.9)*	**<0.001**
2DS middle,%	−21.3 (−22.6, −20.2)	−19.9 (−21.8, −18.3)	**<0.001**	−21.5 (−22.9, −20.3)	−20.0 (−22.0, −18.3)	**<0.001**	−21.0 (−22.0, −19.7)	−19.7 (−21.8, −18.7)	**0.035**
2DS apical,%	–24.0 (−25.8, −23.0)	−23.0 (−24.9, −21.0)	**<0.001**	−23.9 (−26.0, −22.9)	−23.4 (−25.2, −21.0)	**0.005**	−24.5 (−25.5, −23.4)	−22.5 (−24.7, −21.2)	**<0.001**
RVSD4, ms	7.8 (2.9, 11.0)	13.9 (6.7, 20.7)	**<0.001**	7.8 (2.7, 12.1)	9.9 (5.7, 14.0)	0.138	7.2 (3.3, 9.8)	22.7 (20.9, 30.9)**	**<0.001**

Values are the median (25th to 75th quartiles) or the mean ± SD. *P < 0.05, **P < 0.01 compared with RVD-. RA, right atrial; RV, right ventricular; EDA and ESA, end-diastolic and end-systolic areas; FAC, fractional area change; TAM, tricuspid annular motion; *E*, early diastolic velocity; *A*, late diastolic velocity; *s*’, peak systolic velocity with tissue Doppler imaging; *e*’, peak early diastolic velocity with tissue Doppler imaging; IVV, isovolumetric velocity; IVAT, isovolumetric acceleration time; IVA, isovolumetric acceleration; GLS, global longitudinal strain; 2DS, two-dimensional strain (average of septum and free wall); RVSD4, standard deviation in a model with four mid-basal RV segments. Other abbreviations are listed in Table 1. The bolded values mean *p* value < 0.05.

### Hemodynamics in Pulmonary Circulation

In all subjects, the values of PAAT, PAET, PAAT/PAET, PAAT/PEP, and PAV were decreased, whereas PEP, mPAP, and the incidence of TR, TRV, and SPAP were increased following acute HA exposure. Additionally, 25 (23.1%) subjects with Notch were identified at HA. However, subjects with RVD showed higher TRV [259 (231, 293) vs. 236 (218, 263) cm/s, *P* < 0.05] and higher SPAP [30.7 (26.3, 39.4) vs. 27.2 (24.0, 32.7) mmHg, *P* < 0.05] than those without RVD at HA (Table 3 and Figure 3B).

**TABLE 3:** Pulmonary hemodynamic parameters of the participants at sea level and at high altitude.

**Variables**	**Total (*n* = 108)**			**RVD- (*n* = 73)**			**RVD + (*n* = 35)**		
	**SL**	**HA**	***P* value**	**SL**	**HA**	***P* value**	**SL**	**HA**	***P* value**
PAAT, ms	132 ± 16	106 ± 19	<0.001	132 ± 14	106 ± 18	<0.001	131 ± 19	106 ± 19	<0.001
PAET, ms	354 ± 20	343 ± 28	<0.001	357 ± 19	344 ± 28	<0.001	348 ± 21	342 ± 27	0.222
PEP, ms	65 (61, 74)	74 (65, 82)	<0.001	65 (61, 74)	74 (64, 82)	<0.001	64 (61, 74)	74 (67, 82)	0.001
PAAT/PAET	0.37 ± 0.04	0.31 ± 0.05	<0.001	0.37 ± 0.04	0.31 ± 0.05	<0.001	0.38 ± 0.05	0.31 ± 0.05	<0.001
PAAT/PEP	2.00 (1.72, 2.31)	1.40 (1.20, 1.66)	<0.001	1.99 (1.75, 2.31)	1.40 (1.20, 1.69)	<0.001	2.06 (1.62, 2.28)	1.42 (1.20, 1.65)	<0.001
PAV, cm/s	108 (97, 121)	99 (90, 109)	<0.001	105 (97, 121)	98 (90, 108)	<0.001	115 (96, 124)	103 (94, 110)	0.010
mPAP, mmHg	18.5 (14.6, 22.5)	26.3 (21.0, 32.6)	<0.001	18.5 (15.4, 22.5)	24.9 (21.4, 32.3)	<0.001	18.6 (14.0, 22.4)	28.1 (20.2, 32.6)	<0.001
Notch, *n* (%)	0 (0)	25 (23.1)	N/A	0 (0)	19 (26.0)	N/A	0 (0)	6 (17.1)	N/A
TR, *n* (%)	72 (66.7)	86 (79.6)	0.021	49 (67.1)	56 (76.7)	0.197	23 (65.7)	30 (85.7)	0.051
TRV, cm/s	215 (191, 234)	239 (223, 278)	<0.001	215 (187, 235)	236 (218, 263)	<0.001	215 (191, 233)	259 (231, 293)*	<0.001
SPAP, mmHg	23.5 (19.5, 26.8)	27.9 (24.9, 35.8)	<0.001	23.5 (19.0, 27.1)	27.2 (24.0, 32.7)	<0.001	23.4 (19.7, 26.8)	30.7 (26.3, 39.4)*	<0.001

Values are the median (25th to 75th quartiles) or the mean ± SD. *P < 0.05 compared with RVD-. PAAT, pulmonary artery acceleration time; PAET, pulmonary artery ejection time; PEP, pre-ejection period; PAV, peak pulmonary artery velocity; mPAP, mean pulmonary artery pressure; Notch, right ventricular outflow tract systolic Doppler flow velocity envelope; TRV, maximal velocity of the tricuspid regurgitation jet; SPAP, pulmonary systolic pressure. Other abbreviations are listed in Table 1.

### Factors Associated With the Incidence of RVD at HA

The results from univariate logistic regression showed that SaO_2_, RVGLS, and SPAP at HA were associated with the incidence of RVD at HA. Multivariate logistic regression identified two independent factors: SaO_2_ [odds ratio (OR): 0.72, 95% CI: 0.56–0.92; *P* = 0.009] and RVGLS (OR: 1.78, 95% CI: 1.31–2.41; *P* < 0.001) (Table 4).

**TABLE 4:** Logistic regression analysis of clinical factors for the incidence of RVD at high altitude.

**Variables**	**Univariate**		**Stepwise multivariate**	
	**OR (95% CI)**	***P* value**	**OR (95% CI)**	***P* value**
Age	0.99 (0.87, 1.13)	0.890	Not selected	–
Han ethnicity	0.81 (0.24, 2.80)	0.743	Not selected	–
BMI	1.06 (0.85, 1.33)	0.611	Not selected	–
Smoking status	1.07 (0.46, 2.47)	0.877	Not selected	–
HR	1.03 (0.99, 1.07)	**0.126**	-	–
SaO_2_	0.82 (0.69, 0.98)	**0.025**	0.72 (0.56, 0.92)	**0.009**
SBP	1.00 (0.96, 1.04)	0.944	Not selected	–
DBP	1.02 (0.97, 1.06)	0.491	Not selected	–
Hb concentration	0.97 (0.94, 1.01)	**0.172**	-	–
Hematocrit	0.97 (0.90, 1.05)	0.463	Not selected	–
RA EDA	1.08 (0.91, 1.29)	0.352	Not selected	–
RV FAC	1.03 (0.93, 1.15)	0.551	Not selected	–
TAM	0.97 (0.83, 1.13)	0.687	Not selected	–
Tricuspid *E*/*A* ratio	0.66 (0.31, 1.40)	0.280	Not selected	–
Tricuspid *s*’	1.11 (0.79, 1.54)	0.554	Not selected	–
Tricuspid *e*’	0.95 (0.78, 1.17)	0.649	Not selected	–
Tricuspid IVA	0.58 (0.20, 1.69)	0.317	Not selected	–
RVGLS	1.32 (1.10, 1.58)	**0.002**	1.78 (1.31, 2.41)	**< 0.001**
PAET	1.00 (0.98, 1.01)	0.736	Not selected	–
PEP	1.00 (0.96, 1.03)	0.858	Not selected	–
PAV	1.02 (0.99, 1.05)	0.314	Not selected	–
mPAP	1.01 (0.96, 1.06)	0.840	Not selected	–
SPAP	1.07 (1.01, 1.14)	**0.022**	–	–

*Values are the median (25th to 75th quartiles). OR, odds ratio; CI, confidence interval. Other abbreviations are listed in Tables 1–3. The bolded values mean *p* value < 0.05.*

### Associations of mPAP With RVSD4 or RVGLS

**FIGURE 4:**
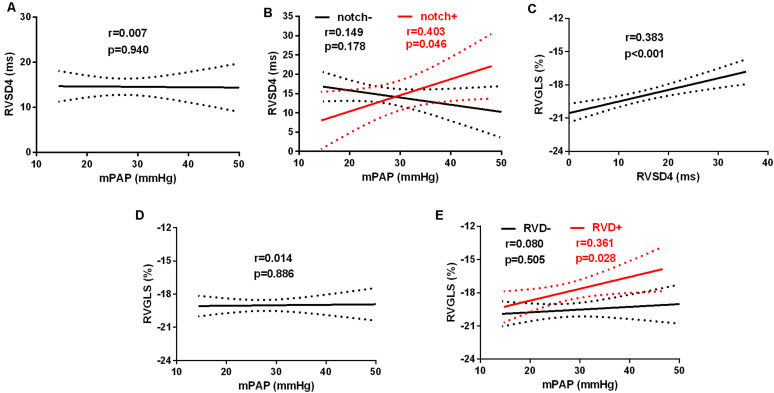
Linear relationships between mPAP and RVGLS or RVSD4 at HA. The linear relationship between mPAP and RVSD4 at HA in **(A)** all subjects and **(B)** subjects with Notch (red) and without Notch (black). The linear relationship between RVGLS and RVSD4 at HA in all subjects **(C)**. The linear relationships between mPAP and RVGLS at HA in **(D)** all subjects and **(E)** subjects with RVD (red) and without RVD (black). Abbreviations are as in Figure 1 and Tables 1, 2.

The linear regression analysis demonstrated that there was no significant correlation between the levels of mPAP and RVSD4 in all subjects at HA (Figure 4A). However, mPAP was linearly associated with RVSD4 at HA in the presence of Notch (*r* = 0.403, *p* = 0.046) (Figure 4B). Furthermore, RVSD4 was linearly associated with RVGLS at HA (*r* = 0.383, *p* < 0.001) (Figure 4C). Although there was no significant correlation between mPAP and RVGLS in all subjects at HA, mPAP was linearly associated with RVGLS at HA in subjects with RVD (*r* = 0.361, *p* = 0.028) (Figures 4D,E).

## Discussion

This is the first study to investigate RVD at HA in healthy subjects and shows that (1) the incidence of RVD after acute HA exposure at 4,100 m is approximately 32.4% in healthy young men; (2) SaO_2_ is an independent determinant of RVD at HA; (3) mPAP is linearly correlated with the magnitude of RVD in the presence of Notch; and (4) the presence and magnitude of RVD at HA result in reduced RVGLS and blunted tricuspid IVA.

### RVD Measurements by STE Analysis

Cardiac magnetic resonance (CMR) is considered a good method for the quantitative assessment of the severity of electromechanical dyssynchrony (Yim et al., 2018). However, unlike portable echocardiography, CMR is not flexible and convenient for field studies at HA. In addition, the lower temporal resolution of CMR compared with echocardiography may affect the analysis of time-to-peak metrics (Schafer et al., 2018). On the other hand, compared to the conventional TDI method, STE analysis can provide some advantages of angle independence and improved signal-to-noise ratio. Moreover, using the longitudinal strain analysis of STE is more accurate than using the wall velocities of TDI for evaluating intraventricular dyssynchrony (Rudski et al., 2010). Additionally, considering the excessive variability in the apical segments for assessing the time-to-peak strain, even in healthy subjects, RVSD4 but not the SD of time-to-peak strain from six segments (RVSD6) was used to estimate RVD (Murata et al., 2017).

### Determinants of HA Exposure-Induced RVD

It has been demonstrated that RVD is mainly determined by the delayed contraction of the RV free wall at basal and mid-segments due to the non-uniformly distributed RV wall stress (Kalogeropoulos et al., 2008). RVD is always associated with elevated RV afterload, especially in patients with different kinds of PH. RVD has been shown to be attributed to an increase in RV afterload (Badagliacca et al., 2015). HA exposure induces a decrease in SaO_2_ as well as an increase in PAP; however, our present results suggest that there are lower levels of SaO_2_ in subjects with RVD and a significant association between SaO_2_ and RVD incidence by univariate and multivariate logistic regression, indicating that HA hypoxia may play a critical role in HA exposure-induced RVD independent of RV overload. Not surprisingly, the absence of RV overload as a contributor to the incidence of RVD may be explained by the fact that the elevated PAP during the first few days at HA was moderate. Consistently, recent results also showed that acute hypoxia for several minutes, but not exercise, could induce regional inhomogeneity (or dyssynchrony) of RV contraction, although both acute hypoxia and exercise led to a comparable increase in RV afterload, suggesting that acute hypoxia was the main determinant of RVD but not RV afterload (Pezzuto et al., 2018). Hypobaric hypoxia is the main characteristic of the HA environment but may result in the reduction in cardiac phosphocreatine (PCr)/ATP in healthy volunteers (Holloway et al., 2011). In an animal model, hypoxia-induced RV dysfunction is attributed to the disturbance of fatty acid oxidation and mitochondrial respiration, which could not be explained by RV overload, suggesting a direct impairment of hypoxia on RV (Gomez-Arroyo et al., 2013). Considering that the RV free wall is very thin and may be more sensitive to HA hypoxia, HA hypoxia-triggered RVD in the present study may be attributed to disturbed energy metabolism. However, the exact mechanisms involved need further investigation.

In our study, RV afterload (mPAP and SPAP) was not associated with the incidence of RVD. Consistently, a previous report showed that patients with borderline PH exhibited marked heterogeneity in RV contraction without any correlation with RV afterload (Lamia et al., 2017). However, our results showed that SPAP was higher in subjects with RVD than in those without RVD and that SPAP was associated with the incidence of RVD by univariate logistic regression, which suggested a weak association between SPAP and RVD. The mPAP in our study was also not a contributor to the development of RVD; however, mPAP was linearly associated with RVSD4 in subjects with Notch formation, which was attributed to pulmonary artery reflected waves under an elevated RV afterload, according to recent guidelines from the American Society of Echocardiography (Bossone et al., 2013), suggesting an underlying relationship between mPAP and RVD. Previous results showed that RV size was another contributing factor to RVD (Hardziyenka et al., 2009). In our study, RV size was not associated with the incidence of RVD, although the RA area and RVEDA had decreased after HA exposure due to hypovolemia, especially after several days of acclimatization, similar to our research setting (Stembridge et al., 2016). Similarly, the elevated afterload seemed to be an important cause of LV dyssynchrony in normal subjects with the absence of other confounding factors, such as ventricular size (Miura et al., 1993).

### RV Function

In our present study, the increases in Hb concentration and hematocrit suggested that the blood volume was significantly decreased, which may lead to the reduction in ventricular filling (Stembridge et al., 2016). Thus, the reduced RV preload due to hypovolemia resulted in a significant but slight decline in RV FAC, which is a load-dependent estimation of RV systolic function (Rudski et al., 2010). Consistent with previous results, although the *P* value is 0.06, the comparable values of FAC with ours showed a trend toward a decline, which may be attributed to the limited sample size (Maufrais et al., 2017). Moreover, the TAM and RV strain also decreased after HA exposure, which was consistent with a previous study (Stembridge et al., 2014). Taken together, these results indicate that although parameters such as FAC and TAM were load dependent, the regional function was altered based on the decreased RV strain.

Although data from PAH patients showed that RVD was strongly correlated with impaired RV function and adverse RV remodeling (Hill et al., 2012), we did not find any additional changes in the decreased FAC and TAM in subjects with RVD. However, subjects with RVD showed lower values of RVGLS than subjects without RVD, which was mainly attributed to the decline in RV strain at basal segments, suggesting that regional RV function was more affected by RVD. Similarly, it has been demonstrated that the presence of RVD was associated with reduced RVGLS in children with PAH as evaluated by CMR (Schafer et al., 2018). Furthermore, we also found a significant increase in tricuspid IVA in all subjects and RVD- subjects but not in RVD + subjects after HA exposure, which indicated that RV IVA was blunted by RVD. It has been suggested that IVA is an ideal method for evaluating acute changes in RV function in a preload- and afterload-independent manner (Vogel et al., 2002). Furthermore, RV IVA has been considered a useful method for identifying early RV systolic alterations and predicting RV contractile dysfunction in the future (Suran et al., 2016). However, whether the blunted RV IVA in subjects with RVD after acute HA exposure persisted or was reversible remains to be investigated.

### Study Limitations

There are some limitations in the present study. First, all the included subjects were young healthy men, and whether the present conclusion could extend to others is still unknown. Second, 2D STE is mainly based on plane selection and therefore may be inferior to 3D STE for evaluating RV function (Smith et al., 2014). Third, echocardiography instead of RV catheterization was used to evaluate the PAP because invasive procedures are unnecessary and unethical in healthy individuals despite right-heart catheterization being the gold standard.

## Conclusion

For the first time, we demonstrated that acute HA exposure could induce RVD in healthy subjects, which may be mainly attributed to the decline in SaO_2_ as well as the increase of RV afterload; the incidence of RVD at HA was associated with reduced RV regional function and blunted myocardial acceleration. Our findings may provide novel insight into the understanding of RV adaptation to HA exposure. Further investigations are needed to clarify the clinical relevance of these findings.

## Data Availability Statement

The datasets generated for this study are available on request to the corresponding author.

## Ethics Statement

The studies involving human participants were reviewed and approved by the Clinical Research Ethics Board at the Third Military Medical University (Army Medical University). The patients/participants provided their written informed consent to participate in this study.

## Author Contributions

LH conceived and designed the study. YY, CL, JT, XD, SB, JY, ZQ, JK, FY, CZ, and RR performed the experiments. YY, CL, and JT analyzed the data and wrote the manuscript. LH critically reviewed the manuscript. All authors approved the final manuscript.

## Conflict of Interest

The authors declare that the research was conducted in the absence of any commercial or financial relationships that could be construed as a potential conflict of interest.

## Footnotes

1 http://www.chictr.org.cn
