# Supplementary material for: Preliminary Study of Right Ventricular Dyssynchrony Under High-Altitude Exposure: Determinants and Impacts
Source: Front Physiol. 2020 Jul 2;11:703. doi: 10.3389/fphys.2020.00703 (PMC7343894; doi:10.3389/fphys.2020.00703)
Supplement: Supplementary file 1 [file Data_Sheet_1.PDF]

Supplemental Table 1. ICC analysis of intra- and interobserver variations.

Variables	Intraobserver variation		P value	Interobserver variation		P value
	ICC	95%CI		ICC	95%CI	
RVEDA, cm^2	0.986	0.965-0.994	<0.001	0.993	0.982-0.997	<0.001
RVESA, cm^2	0.978	0.944-0.991	<0.001	0.972	0.928-0.989	<0.001
Tricuspid E, cm/s	0.989	0.972-0.996	<0.001	0.967	0.918-0.987	<0.001
Tricuspid A, cm/s	0.961	0.903-0.984	<0.001	0.954	0.885-0.982	<0.001
Tricuspid s’, cm/s	0.972	0.931-0.989	<0.001	0.944	0.858-0.978	<0.001
Tricuspid e’, cm/s	0.910	0.778-0.964	<0.001	0.878	0.691-0.952	<0.001
Tricuspid IVV, cm/s	0.902	0.756-0.961	<0.001	0.925	0.811-0.970	<0.001
Tricuspid IVAT, ms	0.853	0.635-0.941	<0.001	0.901	0.749-0.961	<0.001
RVGLS, %	0.923	0.800-0.971	<0.001	0.819	0.516-0.932	<0.001
2DS basal, %	0.916	0.734-0.974	<0.001	0.818	0.496-0.934	0.001
2DS mid, %	0.826	0.514-0.938	0.001	0.838	0.518-0.946	0.001
2DS Apical, %	0.849	0.604-0.943	<0.001	0.827	0.461-0.944	0.002
Mean T2P, ms	0.889	0.708-0.958	<0.001	0.880	0.668-0.956	<0.001
PAAT, ms	0.873	0.685-0.949	<0.001	0.987	0.967-0.995	<0.001
PAET, ms	0.967	0.918-0.987	<0.001	0.974	0.935-0.990	<0.001
PEP, ms	0.949	0.847-0.980	<0.001	0.917	0.791-0.967	<0.001
PAV, cm/s	0.980	0.950-0.992	<0.001	0.992	0.979-0.997	<0.001
TRV, cm/s	0.969	0.924-0.988	<0.001	0.971	0.927-0.989	<0.001

Abbreviations as in Table 2 and 3.
